# Diffusion spectrum imaging in patients with idiopathic normal pressure hydrocephalus: correlation with ventricular enlargement

**DOI:** 10.1186/s12883-024-03741-w

**Published:** 2024-07-16

**Authors:** Qian Wu, Wenjie He, Chenyuan Liu, Xiaolin Yang, Jiakuan Chen, Boyan Xu, Xi Zhou, Guodong Huang, Jun Xia

**Affiliations:** 1 Department of Radiology, Shenzhen Second People’s Hospital, The First Affiliated Hospital of Shenzhen University, Shenzhen University, 3002 SunGang Road West, Shenzhen, Guangdong Province 518035 China; 2 Five-year Clinical Medicine, Xiangya School of Medicine, Central South University, Changsha, Hunan Province 410083 China; 3 Longgang Central Hospital of Shenzhen, Shenzhen, China; 4 Department of Radiology, South China Hospital, Medical School, Shenzhen University, Shenzhen, 518116 China; 5 MR Research, GE Healthcare, Beijing, 100076 China; 6 Department of Neurosurgery, Shenzhen Second people’s hospital, The First Affiliated Hospital of Shenzhen University, Shenzhen University, 3002 SunGang Road West, Shenzhen, Guangdong Province 518035 China

**Keywords:** Idiopathic normal pressure hydrocephalus, White matter, Diffusion spectrum imaging

## Abstract

**Background:**

To investigate the association between white matter changes and ventricular expansion in idiopathic normal pressure hydrocephalus (iNPH) based on diffusion spectrum imaging (DSI).

**Methods:**

We included 32 patients with iNPH who underwent DSI using a 3T MRI scanner. The lateral ventricles were manually segmented, and ventricular volumes were measured. Two methods were utilised in the study: manual region-of-interest (ROI) delineation and tract diffusion profile analysis. General fractional anisotropy (GFA) and fractional anisotropy (FA) were extracted in different white matter regions, including the bilateral internal capsule (anterior and posterior limbs) and corpus callosum (body, genu, and splenium) with manual ROI delineation. The 18 main tracts in the brain of each patient were extracted; the diffusion metrics of 100 equidistant nodes on each fibre were calculated, and Spearman’s correlation coefficient was used to determine the correlation between diffusion measures and ventricular volume of iNPH patients.

**Results:**

The GFA and FA of all ROI showed no significant correlation with lateral ventricular volume. However, in the tract diffusion profile analysis, lateral ventricular volume was positively correlated with part of the cingulum bundle, left corticospinal tract, and bilateral thalamic radiation posterior, whereas it was negatively correlated with the bilateral cingulum parahippocampal (all *p* < 0.05).

**Conclusions:**

The effect of ventricular enlargement in iNPH on some white matter fibre tracts around the ventricles was limited and polarizing, and most white matter fibre tract integrity changes were not associated with ventricular enlargement; this reflects that multiple pathological mechanisms may have been combined to cause white matter alterations in iNPH.

**Supplementary Information:**

The online version contains supplementary material available at 10.1186/s12883-024-03741-w.

## Background

Idiopathic normal pressure hydrocephalus (iNPH), a neurological disorder of unknown aetiology, can be treated surgically and occurs mainly in elderly individuals [1]. Patients present with ventricular enlargement detected via radiological assessment with a normal cerebrospinal fluid (CSF) pressure range [2]. iNPH is associated with various white matter abnormalities; the corticospinal tract (CST) and corpus callosum are significantly associated with gait and cognitive impairment, respectively [3, 4]. Ventricular dilatation compresses and stretches the white matter and corpus callosum nerve fibre bundles, resulting in physiological dysfunction and partial symptom recovery after CSF shunt surgery [5].

Previous studies have used diffusion tensor imaging (DTI) to investigate subcortical involvement in the brains of patients with iNPH and have attempted to demonstrate changes in white matter integrity [3]. However, DTI cannot be used to evaluate the crossing and twinning of fibre bundles because it depends on the Gaussian parameterisation of diffusion [6]. Diffusion spectrum imaging (DSI) is a relatively new magnetic resonance diffusion imaging technique that can noninvasively detect complex intracranial white matter fibre structures and fibre bundle structural alterations. It can demonstrate fibre crossings, tangles, and disruptions to some small fibre structures more accurately than conventional DTI [7]. Similar to fractional anisotropy (FA) in DTI, generalised fractional anisotropy (GFA) is the main DSI quantitative parameter and represents the directional coherence of water molecule diffusion [8]. The region-of-interest (ROI) method is commonly used to study white matter fibre microstructural alterations in iNPH based on DTI. However, ROI analysis is slightly rough, can only be performed on specific brain regions, and cannot accurately reflect changes in every fibre bundle. The tract diffusion profile analysis proposed by Yeatman et al. [9] can automatically track and extract the whole brain’s major fibre tracts and divide them into 100 nodes, thus quantifying the fibre tracts at the microscopic level. DSI is mostly applied to mental disorders [10, 11] and Alzheimer’s disease [12] but is rarely used in iNPH to explore white matter alterations. Therefore, in the early stages, we used tract diffusion profile analysis based on DSI to assess changes in white-matter integrity across iNPH and normal control groups, and found that most of the fasciculus abnormalities in iNPH were relatively confined to specific areas. Yang et al. [13] also found that different sections of the same fasciculus exhibited varying diffusion alterations. However, the relationship between major white matter fibre tracts and ventriculomegaly in the whole brain remains unclear.

We hypothesised that ventricular expansion stretches and compresses the periventricular white matter and affects the deep white matter, causing impaired white matter fibre integrity. Therefore, we aimed to investigate the relationship between white matter integrity and ventricular expansion in iNPH based on DSI using two methods --- manual ROI delineation and tract diffusion profile analysis.

## Methods

### Participants

**Fig. 1:**
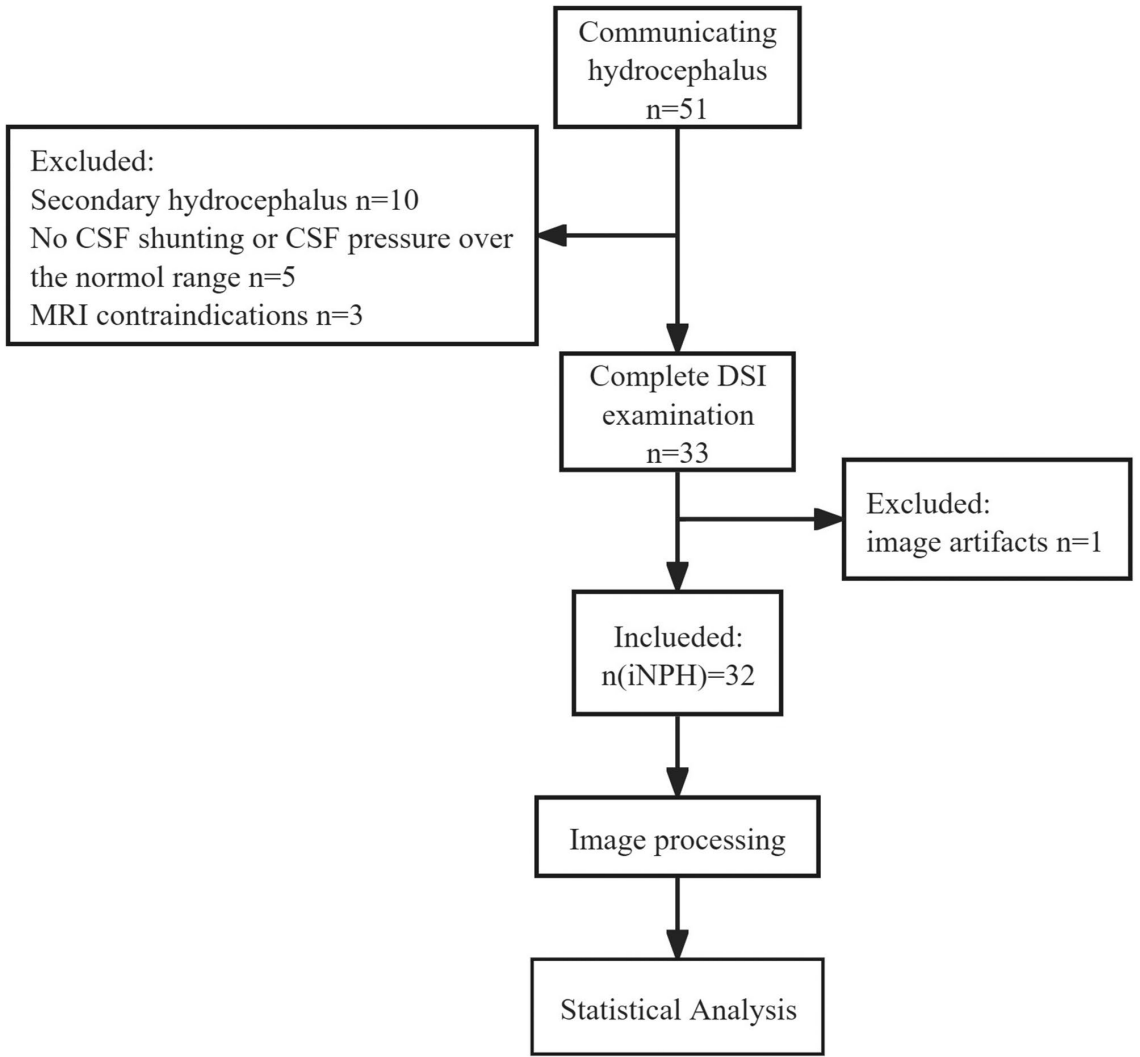
Study flowchart of the inclusion criteria for iNPH participants

This study included 32 patients (11 men, 22 women; mean age ± standard deviation, 67.9 ± 9.2 years) with iNPH, defined according to the relevant guidelines [1], who were treated in the neurosurgery department of our hospital between January 2019 and March 2022. The study flowchart for the participants’ inclusion and exclusion is illustrated in Fig. 1. In the early stage of the study, fifty-one patients were included by a neurologist with 10 years of experience based on the following criteria: (1) older than 60 years of age; (2) presenting with one or more other typical triad symptoms (gait disturbance, cognitive impairment, and urinary incontinence), which were measurable on the iNPH grading scale (iNPHGS) [14]; and (3) ventriculomegaly with an Evan’s index > 0.3, indicated by short-term radiological imaging examinations. Nineteen of these patients were excluded because of secondary hydrocephalus (with a clear history of neurologic tumors, cerebral haemorrhage or ischaemic stroke, intracranial infection, or trauma; *n* = 10), absence of CSF shunting or CSF pressure exceeding the normal threshold (*n* = 5), MRI contraindications (*n* = 3), or image artefacts (*n* = 1). All recruited patients underwent the same MRI protocol and assessment of clinical symptoms according to the iNPHGS scale and the modified Rankin Scale (mRS) [15] before CSF shunting. This research was approved by the local institutional review board (approval number 2023-277-01PJ) and conducted ethically following the World Medical Association’s Declaration of Helsinki.

### MRI data collection

All subjects underwent MRI examination on a 3-Tesla MR scanner (Prisma; Siemens; Erlangen, Germany) with 20-channel phase-array head coils. The participants’ heads were to remain stationary during the scanning as much as possible or with the help of a fixator if necessary. All 32 participants were scanned using the same protocol. The MRI session comprised 3D sagittal T1-weighted sequences and a DSI sequence. Additionally, we obtained pulsed-gradient, twice-refocused, and spin-echo planar imaging sequences. The T1W acquisition parameters were as follows: repetition time/echo time = 2300/3.55 ms; flip angle, 8°; slice thickness = 0.9 mm; field of view (FOV) = 240 mm × 240 mm; and matrix = 256 × 256. The DSI collection parameters were as follows: repetition time/echo time = 6300/71 ms; slice thickness = 2.2 mm; number of sections on the horizontal axis = 60; FOV = 220 mm × 220 mm; matrix = 100 × 100; total diffusion sampling = 128; and maximum diffusion sensitivity (b-values max) = 3000 s/mm^2^.

### Anatomical and diffusion data pre-processing

The pre-processing of the diffusion and anatomical data was performed using the methodology of another previous DSI study by our team. According to the research methodology described by Yang et al. [13], anatomical and diffusion data were pre-processed using QSIPrep version 0.12.1 based on Nipype 1.5.1 [16]. The T1W image was corrected for intensity non-uniformity using N4BiasFieldCorrection (ANTs 2.3.1) [17] and used as a T1W reference throughout the workflow. The T1W reference was then skull-stripped using antsBrainExtraction.sh (ANTs2.3.1), with OASIS as the target template. Spatial normalisation was achieved by registering the extracted T1W brain volume to the ICBM 152 nonlinear asymmetrical template version 2009c using antsRegistration (ANTs 2.3.1). Brain tissue segmentation was performed on the extracted T1W images using FAST (FSL 6.0.3: b862cdd5).

The initial motion correction was performed using the b = 0 images and an unbiased b = 0 template was constructed. Then, the head motion in the b > 0 images was estimated and image reconstruction was conducted using the SHORELine method [18]. Confounding time series were calculated based on the pre-processed diffusion-weighted images (DWI) [19]. Finally, the DWI time series was resampled to the anterior commissure-posterior commissure (ACPC) to generate a pre-processed DWI run in the ACPC space.

### Tractography and automatic tract recognition

In our team’s preliminary study of DSI in iNPH, we automatically extracted white matter fibres from the brains of patients with iNPH and calculated the diffusion parameters of 100 equidistant nodes along each fibre to quantify and evaluate integrity changes in different segments of the tracts. Similarly, after pre-processing, diffusion data were reconstructed using generalised q-sampling imaging [20] in DSI Studio software (http://dsistudio.labsolver.org). According to the method described by Yang et al. [13], augmented fibre tracking was used for automatic track recognition [21] and a deterministic fibre tracking algorithm [22] was used to improve reproducibility. Eighteen major fibres (including some subcomponents) were mapped using the anatomy prior to introducing a tractography atlas [23] with a distance tolerance of 16 mm. Topology-informed pruning [24] was applied to remove false connections. After visual verification of the automatically recognised fibre tracts, we uniformly sampled 100 equidistant points along each tract and obtained two diffusion metrics, GFA and FA. The analysed tracts included association, commissural, and projection fibres, whose directions were regulated as anterior to posterior, right to left, and inferior to superior, respectively. These fibres included the corpus callosum forceps minor, corpus callosum forceps major, bilateral arcuate fasciculus (AF), corticospinal tract (CST), cingulum few, uncinate fasciculus (UF), superior longitudinal fasciculus (SLF), thalamic radiation (TR), inferior longitudinal fasciculus, and inferior fronto-occipital fasciculus. According to the updated HCP1065 atlas [23], three subcomponents of the SLF, SLF 1, 2, and 3, were extracted and analyzed. Similarly, the identified bilateral cingulum was subdivided into the bilateral cingulum frontal parahippocampal (C_FPH), cingulum frontal parietal (C_FP), cingulum parahippocampal parietal (C_PHP), cingulum parahippocampal (C_PH), and cingulum rarolfactory; the bilateral TR extracted included the bilateral thalamic radiation anterior (TRA), thalamic radiation posterior (TRP), and thalamic radiation superior (TRS).

### Region-of-interest analysis

**Fig. 2:**
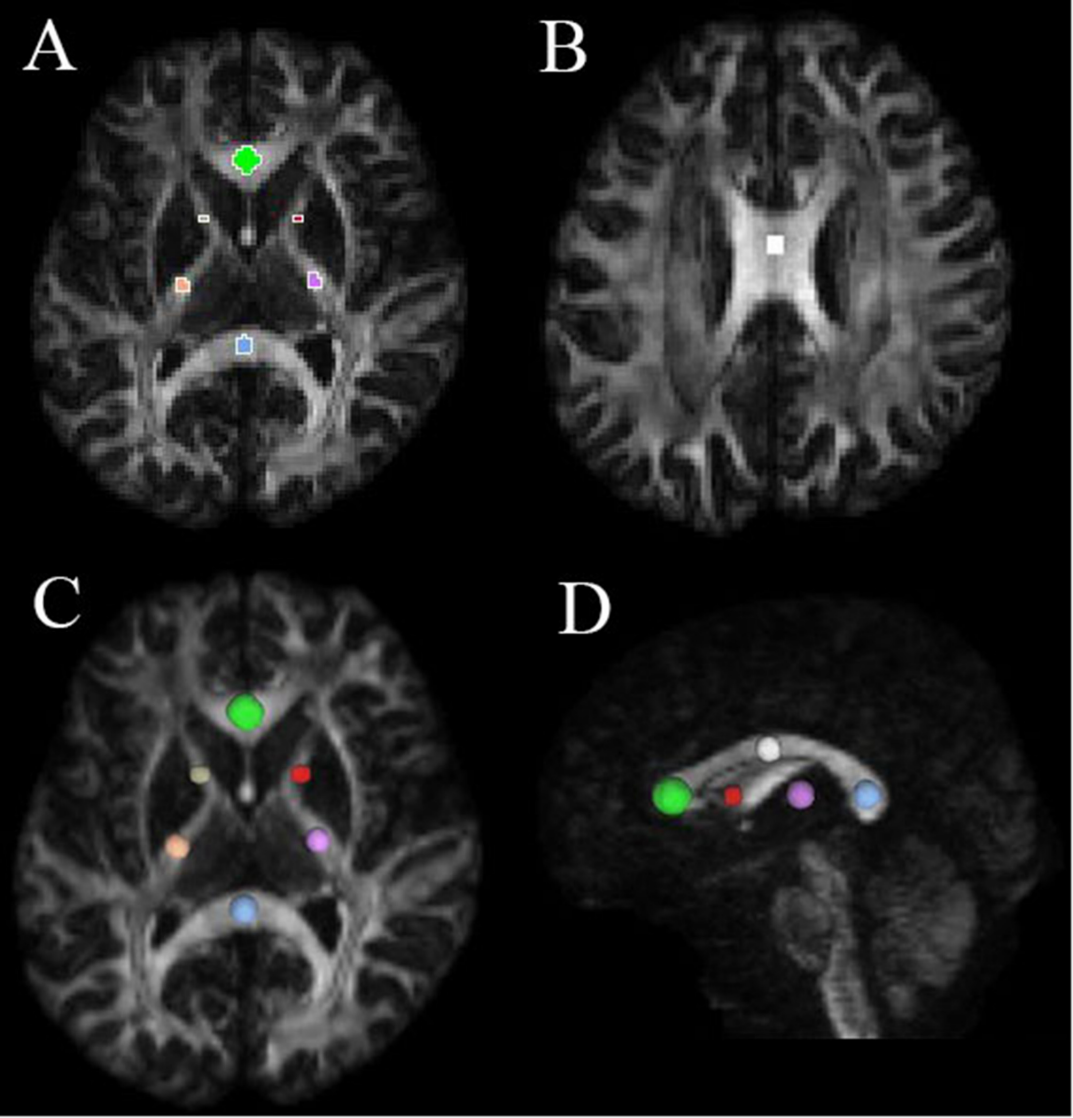
Seven regions of interests (ROIs) placed along the genu, body, and splenium of the corpus callosum (CC), right and left anterior limb of the internal capsule (IC), and right and left posterior limb of the IC; **(A)** green, blue, grey, red, pink, and purple indicate the ROIs of the genu of the CC, splenium of the CC, right and left anterior limb of the IC, and right and left posterior limb of the IC, respectively; **(B)** white indicates the ROI of the body of the CC; (**C** and **D**) spatial projection of the seven ROIs on brain regions

To measure DSI parameters in different white matter regions in patients with iNPH, we used DSI Studio software (http://dsi-studio.labsolver.org/), an accurate traction method, to compute and plot the DSI image data for analysis. The ROIs were drawn manually in the corpus callosum (genu, body, and splenium) of all participants with volumes of 333.8 mm^3^, 88.6 mm^3^, and 139.6 mm^3^, respectively. The ROIs were outlined bilaterally and symmetrically in the right and left internal capsule (IC) (anterior and posterior limb) of all participants with the same size and shape on both sides, with volumes of 40.9 mm^3^ and 98.8 mm^3^, respectively (Fig. 2). A neuroradiologist with 15 years of experience delineated all the ROIs. The mean GFA and FA values for each ROI were calculated for statistical analysis.

### Ventricular enlargement assessment

**Fig. 3:**
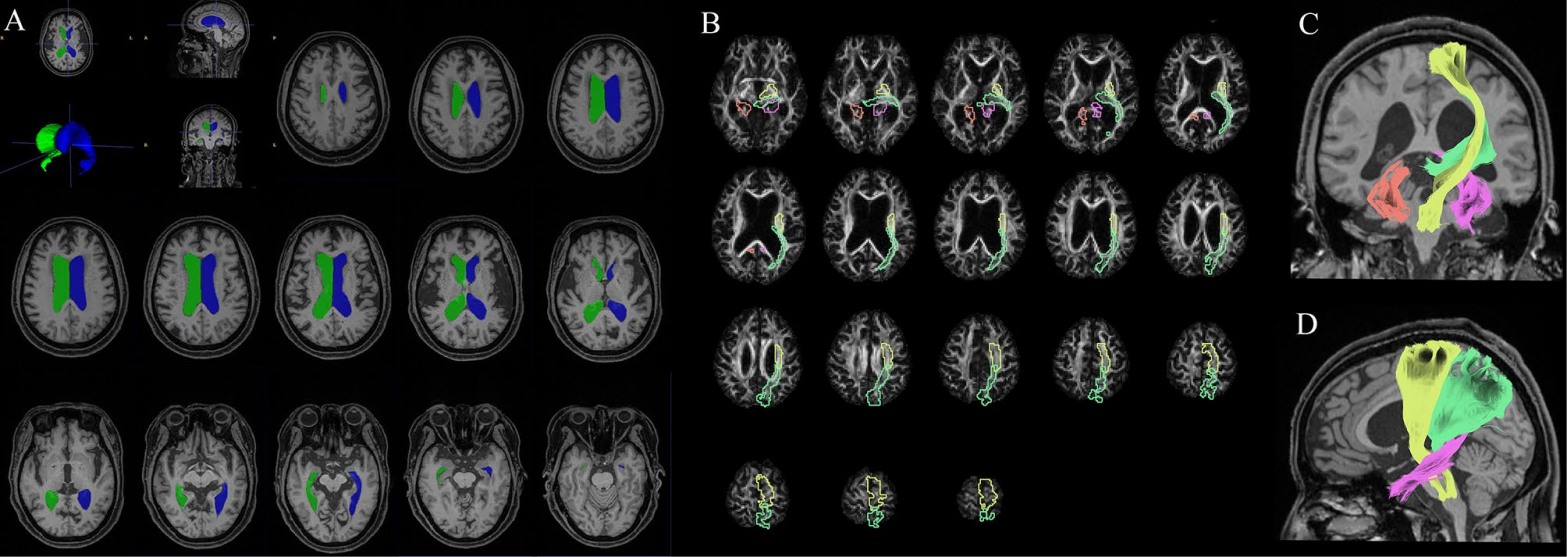
Measurement of LVV and GFA maps of a typical iNPH patient and the reconstruction results of bilateral C_PH, left CST, and left TRP. **(A)** Measurement of LVV of a typical iNPH patient. Green and blue indicate the right and left lateral ventricles, respectively. (**B**, **C**, and **D**) GFA maps and reconstruction results of bilateral C_PH, left CST, and left TRP of a typical iNPH patient. **(B)** Colour bar from 0 (black) to 0.2 (white); red, purple, yellow, and green indicate the range of the right C_PH, left C_PH, left CST, and left TRP, respectively. (**C** and **D**) Reconstruction results of the bilateral C_PH, left CST and left TRP

ITK-SNAP open-source software (v3.6.0-RC1; http://www.itksnap.org) was used to measure the lateral ventricular volume (LVV), which was determined using digital imaging and inference from patients’ medical images. According to the 3D thin-layer T1W imaging that contained the ventricles, we used the ‘freehand drawing style—polygon’ of the ‘polygon inspector’ modules to manually paint out the left and right lateral ventricles. After segmenting and modelling the ventricle, the volume information was calculated automatically (Fig. 3). Two independent operators (a radiologist and a neurosurgeon) measured the LVVs. The intraclass correlation coefficient for the LVV was 0.98.

### Statistical analysis

Statistical Package for the Social Sciences (SPSS) 25.0 software was used to perform the statistical analysis of clinical and demographic variables. The Wilcoxon signed-rank test was used to evaluate differences in the parameters (FA and GFA) for certain ROIs between the left and right hemispheres. Spearman’s correlation coefficient was then used to estimate the relationship between the LVV and ROI diffusion metrics. For tract diffusion profile analysis, point-wise statistical analyses were conducted using the SciPy package (version 1.9.0, https://scipy.org/). Spearman’s correlation coefficient was used to determine the correlation between the LVV and diffusion metrics of patients with iNPH along each tract for 100 nodes. Significance was corrected using permutation-based multiple comparison corrections [25], yielding a family-wise error (FWE) significant cluster size. A tract segment was considered significant if the number of adjacent points (*p* < 0.05, uncorrected) was equal to or larger than the cluster size determined by FWE.

## Results

### Demographics and clinical information

The demographics and clinical information of the study participants were presented in Table 1.

**Table 1:** Demographics and clinical information of study participants with iNPH

	iNPH (*N* = 32)
Age (years)	67.9 ± 9.2
Sex (male: female)	11:22
EI	0.34 ± 0.03
LVV (ml)	124.33 ± 49.70
Total iNPHGS	4.6 ± 2.7
mRS	2.6 ± 0.3

Values are expressed as the mean ± standard deviation

Abbreviations: iNPH, idiopathic normal pressure hydrocephalus; EI, Evan’s index; lateral ventricular volume (LVV); iNPHGS, iNPH grading scale; mRS, modified Rankin Scale

### Associations of FA/GFA of white matter and LVV

#### ROI analysis

The FA and GFA were significantly higher in the IC’s left anterior and posterior limbs than in the opposite side (Table 2). No significant correlation was observed between white matter diffusion parameters and LVV in all brain regions, with the body of the corpus callosum differing from the other regions in that its reduced white matter integrity was negatively correlated with LVV, although the p-value was not significant (r _FA_ = -0.109, *p* > 0.05, r _GFA_ = -0.126, *p* > 0.05) (Table 3).

**Table 2:** Mean diffusion metrics (FA and GFA) of 7 ROIs

ROIs	FA		GFA		*p*-value*	
	L	*R*	L	*R*	FA	GFA
ALIC	0.660 ± 0.067	0.648 ± 0.063	0.057 ± 0.008	0.050 ± 0.007	< 0.05	< 0.05
PLIC	0.637 ± 0.059	0.627 ± 0.056	0.070 ± 0.011	0.064 ± 0.009	< 0.05	< 0.05
Genu of CC	0.486 ± 0.074		0.079 ± 0.007		-	
Body of CC	0.579 ± 0.076		0.077 ± 0.009		-	
Splenium of CC	0.379 ± 0.062		0.081 ± 0.007		-	

Abbreviations: ROI, region-of-interest; FA, fractional anisotropy; GFA, general fractional anisotropy; ALIC, anterior limb of internal capsule; PLIC, posterior limb of internal capsule; CC, corpus callosum; L, left; R, right; *left vs. right; -, no statistical analysis

**Table 3:** Correlations between bilateral ventricular volume and mean diffusion metrics (FA and GFA) of ROIs

ROIs	Diffusion metrics	Lateral ventricular volume	
		Correlation coefficient (r)	*p*-value
Genu of CC	FA	0.113	0.536
	GFA	-0.041	0.823
Body of CC	FA	-0.109	0.553
	GFA	-0.126	0.494
Splenium of CC	FA	0.310	0.084
	GFA	0.010	0.586
ALIC_L	FA	0.168	0.356
	GFA	0.191	0.295
ALIC_R	FA	0.200	0.271
	GFA	0.310	0.085
PLIC_L	FA	0.250	0.168
	GFA	0.052	0.777
PLIC_R	FA	0.281	0.119
	GFA	0.126	0.491

*p < 0.05; **p < 0.01

Abbreviations: ROI, region-of-interest; GFA, general fractional anisotropy; FA, fractional anisotropy; CC, corpus callosum; ALIC, anterior limb of internal capsule; PLIC, posterior limb of internal capsule; L, left; R, right

#### Tract diffusion profile analysis

**Fig. 4:**
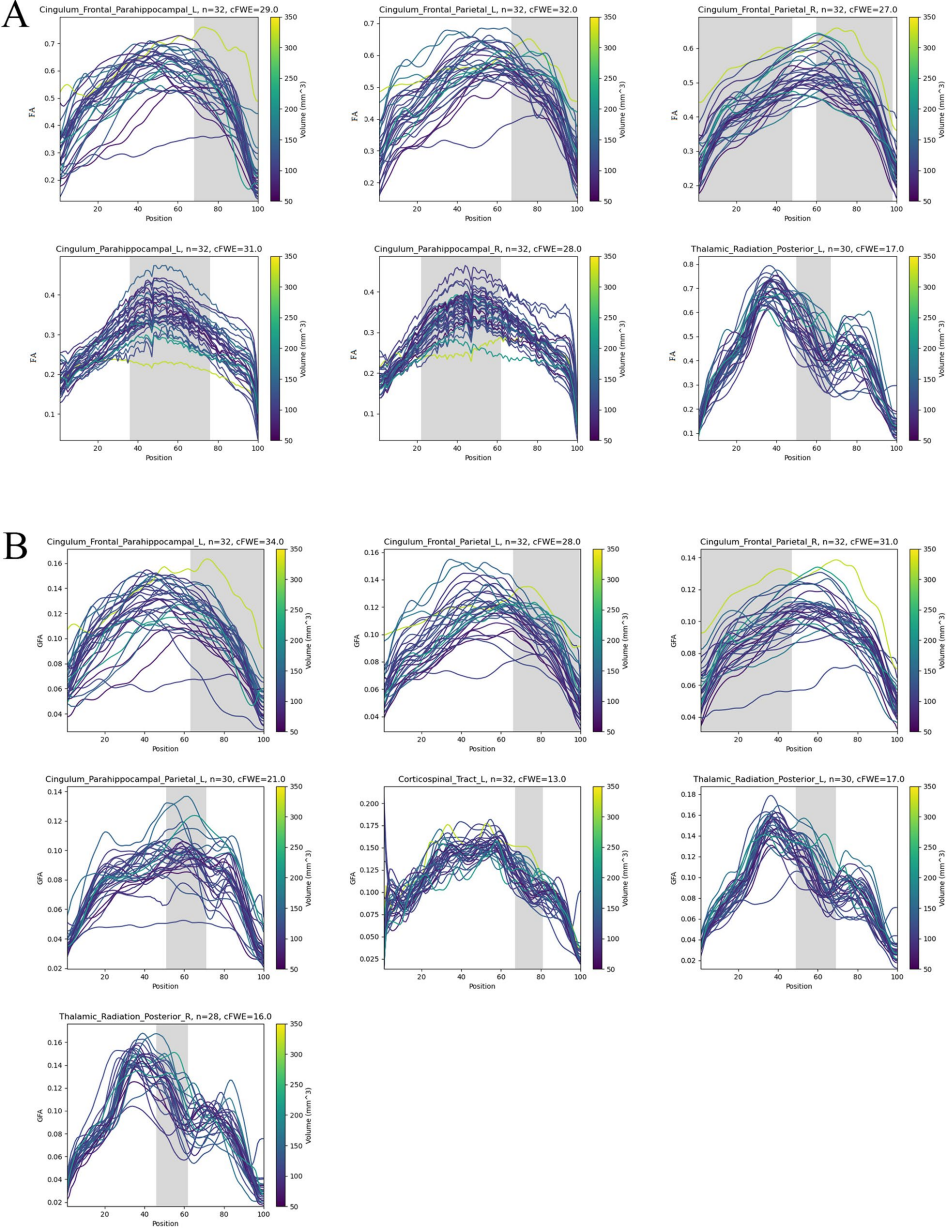
Pointwise correlation analysis of the FA or GFA of certain fibres and the lateral ventricular volume in iNPH. Each line represents the participant analysed, different line colours indicate different lateral ventricular volumes. Colour bars from 50 ml (purple) to 350 ml (yellow), and grey-shaded parts are positions with significant p-values in correlation analysis (family-wise error correction, *p* < 0.05) in the analysed fibre variables in iNPH. **(A)** Segmental FA of fibre tracts was correlated with lateral ventricular volume, including the cingulum frontal parahippocampal, cingulum frontal parietal, cingulum parahippocampal, and thalamic radiation posterior. **(B)** Segmental GFA of fibre tracts was correlated with lateral ventricular volume, including the cingulum frontal parahippocampal, cingulum frontal parietal, cingulum parahippocampal parietal, corticospinal tract, and thalamic radiation posterior. Abbreviations: iNPH, idiopathic normal pressure hydrocephalus; L, left; R, right; FA, fractional anisotropy; GFA, general fractional anisotropy; n (iNPH), successfully recognized number of patients with iNPH in these fibres

We included 31 fibre bundles, including their subcomponents, in the subsequent statistical analysis, with an identification rate higher than 70% (Supplementary Material 1). Point-wise correlation analyses between the bilateral ventricular volume and the FA and GFA of fibre tracts were performed, and the positive results are illustrated in Fig. 4 (The remaining fibre bundle correlation analysis results are in Supplementary Material 2). Local segmental diffusion parameters were correlated with the LVV for all fibre bundles, and most segments were located in the middle part of these tracts (FWE correction, *p* < 0.05). To further evaluate the degree of linear correlation between these fibre tracts in Fig. 4 and LVV, the mean FA and GFA of these tract segments was calculated for correlation analysis with LVV, and the results are presented in Table 4. All local mean diffusion parameters (FA or GFA) of the cingulum were positively correlated with bilateral ventricular volume (*p* < 0.05), except for bilateral C_PH, which was negatively correlated with LVV (r _FA_ = -0.504 and r _FA_ = -0.467, respectively; *p* < 0.05). Both the left CST (r _GFA_ = 0.589, *p* < 0.01) and the bilateral TRP (left, r _FA_ = 0.523 and r _GFA_ = 0.654, respectively; right, r _GFA_ = 0.588; all *p* < 0.05) were positively and significantly correlated with LVV.

**Table 4:** Correlations between ventricular volume and mean diffusion metrics of abnormal segment in certain fasciculi

Tract	Significant segments	Lateral ventricular volume
C_FPH_L		
FA	68–100	0.576^**^
GFA	63–100	0.523^*^
C_FP_L		
FA	67–100	0.686^**^
GFA	66–100	0.691^**^
C_FP_R		
FA	0–48 59–97	0.534^*^
GFA	1–47	0.473^*^
C_PH_L		
FA	36–76	-0.504^*^
C_PH_R		
FA	22–62	-0.467^*^
C_PHP_L		
GFA	51–71	0.423^*^
CST_L		
GFA	67–81	0.589^**^
TRP_L		
FA	50–67	0.523^*^
GFA	49–69	0.654^**^
TRP_R		
GFA	46–62	0.588^**^

Spearman’s significant correlations are listed; values are expressed as correlation coefficient (r); *p < 0.05; **p < 0.01

Abbreviations: C_FPH, cingulum frontal parahippocampal; C_FP, cingulum frontal parietal; C_PH, cingulum parahippocampal; C_PHP, cingulum parahippocampal parietal; CST, corticospinal tract; TRP, thalamic radiation posterior; FA, fractional anisotropy; GFA, general fractional anisotropy; L, left; R, right; -, no statistical analysis

## Discussion

We used two DSI-based study methods, manual ROI delineation and tract profile analysis, to elucidate the relationship between white matter integrity and LVV in patients with iNPH. Our findings indicated that the integrity of specific fibre segments in the paraventricular area were correlated with ventricular volume by tract diffusion profile analysis, and that the majority of white matter fibre integrity did not correlate with ventricle enlargement. Furthermore, tract profile analysis could make up for shortcomings of manual ROI delineation in quantitatively evaluating the relationship between ventricular enlargement and the integrity of each fibre fasciculus.

The ROI method is widely used to study microstructural changes in white matter fibres in iNPH [26, 27]; and although the ROI method is easily accessible to clinicians, it could be subjective. The method for outlining the ROIs in different brain regions is drawn manually, which is time- and energy-consuming and introduces a degree of subjectivity, leading to low reproducibility. Additionally, owing to multiple fibre tracts crossing a voxel, calculating the average FA or GFA within a region does not reflect the actual fibre tracts influenced by ventricular dilatation. The analysis of white matter in different brain regions using the ROI method is not easily achieved; therefore, we used tract diffusion profile analysis to automatically track and extract the 18 major fibre tracts and their subcomponents in the whole brain. We divided the tracked fibre tracts into 100 nodes for statistical analysis at the microscopic level, thus enabling the localisation and quantification of fibre bundle abnormalities. Tract diffusion profile analysis uses a deterministic fibre tracking algorithm to complete fibre bundle tracking. It employs a rigorous tractography atlas to modify and remove erroneous bundles with a very high degree of reproducibility to achieve an accurate quantitative assessment of each fibre bundle [9]. Therefore, this could explain why the ROI analysis presented negative results, whereas the tract profile analysis did not, particularly in the posterior limb region of the IC where the CSTs travel. These inconsistencies are most likely caused by a flaw in the ROI methodology itself.

Our team explored white-matter abnormality patterns in iNPH compared to controls along with tract analysis based on DSI, such as the coexistence of increased and decreased FA or GFA in different segments of CST and TRP, as well as the decreased FA or GFA and impaired fibre integrity throughout C_PH [13]. In this study, the effect of ventricular enlargement on periventricular white matter fibre tracts was polarising; that is, the FA or GFA of certain fibre tracts increases or decreases simultaneously with ventriculomegaly, and FA values heterogeneity may be related to the coexistence of two different pathological processes: neurodegeneration (decreased FA) and ventricular expansion (increased FA) [28]. GFA is similar to FA and reflects changes in fibre tract integrity. In the brain, FA values are influenced by the diameter, density, and nerve fibre myelin formation extent. According to previous studies, enlarged lateral ventricles mechanically compress axons in the CST, causing water molecules to align along the axons, which leads to increased FA values [29, 30]. Our finding of elevated FA or GFA in the localised left CST and bilateral TRP segments with increasing ventricular volume confirmed this. The CST projects fibres through the posterior limb of the IC, through which the CST is connected to the cerebral cortex and controls somatic movements. The thalamic radiation posterior comprises fibre tracts from the thalamus to the occipital lobe, the local anatomical arrangement of which is closely related to the lateral ventricle [23].

Additionally, we discovered that part of the cingulum bundle was positively correlated with the ventricular volume, which means that the greater the ventricular volume, the stronger the compression and stretching of the fibre bundle and the corresponding increase in FA or GFA. The cingulum bundle travels next to the brain’s midline above the corpus callosum. In patients with iNPH, the enlarged and elevated lateral ventricle, and the dilated Sylvian fissures narrowed the callosal angle. With the characteristic imaging manifestations of iNPH, including the disproportionately enlarged subarachnoid space hydrocephalus pattern defined by narrowed high convexity and tight medial parietal sulci [1], we speculate that the above morphological changes produced compression and increased the FA or GFA of the cingulum bundle.

Studies have revealed that as the disease progresses, mechanical stress may lead to axonal atrophy and irreversible damage [31], resulting in a corresponding decrease in FA values. This may explain the negative correlation between changes in the integrity of the bilateral C_PH and ventricular enlargement. Similarly, Tan et al. [32] discovered reduced FA values in the right hippocampal gyrus of patients with iNPH and hypothesised that this was associated with increased bilateral ventricular volume, a part of the hippocampal formation that lies axially below the splenium of the corpus callosum. Hippocampal structures play an important role in learning and memory. Reduced C_PH fibre tract integrity occurs in patients with iNPH, possibly owing to irreversible axonal damage to neurons caused by chronic ventricular dilatation-induced long-term mechanical stress, which may be related to certain cognitive dysfunctions in iNPH.

This study had some limitations. First, this was a cross-sectional study with a small sample size, and all patients were recruited at one centre; therefore, these results should be interpreted cautiously. Further studies are needed to confirm our results, and future studies are needed to investigate the clinical relevance of cross-tract changes in these diffusion parameters in iNPH. Second, patients with iNPH have greatly enlarged ventricles, which could have a technical impact on fibre tracking results and the observed abnormalities in bundle analysis. Pre-processing and analysis tools have been validated [16]; however, additional errors induced by the enlarged ventricles should be further elucidated. Strict criteria are typically used in automated fibre identification to remove erroneous fibre bundles and ensure the identified fibres’ correctness, which is another reason for fibre identification failure [33]. Enhanced fibre tracking should be further adapted to improve the accuracy in patients with iNPH.

## Conclusions

In summary, we utilised DSI to clarify the relationship between cerebral white matter integrity changes and ventricular expansion in patients with iNPH. We discovered that the effect of ventricular enlargement in iNPH on some white matter fibre tracts around the ventricles was limited and polarising, and most white matter fibre tract integrity changes were not associated with ventricular enlargement. Therefore, we speculated that multiple pathological processes may have been combined to cause white matter alterations in iNPH, and further studies are needed to elucidate them.

### Electronic supplementary material

Below is the link to the electronic supplementary material.

Supplementary Material 1

Supplementary Material 2

## Data Availability

No datasets were generated or analysed during the current study.

## Abbreviations

iNPH: idiopathic normal pressure hydrocephalus
DSI: Diffusion spectrum imaging
ROI: Region-of-interest
GFA: General fractional anisotropy
FA: Fractional anisotropy
CSF: Cerebrospinal fluid
CST: Corticospinal tract
DTI: Diffusion tensor imaging
DWI: Diffusion-weighted images
SLF: Superior longitudinal fasciculus
TRP: Thalamic radiation posterior
LVV: Lateral ventricular volume
FEW: Family-wise error
IC: Internal capsule
MRI: Magnetic resonance imaging
CPH: Cingulum parahippocampal
C_FPH: Cingulum frontal parahippocampal
C_FP: Cingulum frontal parietal
C_PHP: Cingulum parahippocampal parietal
