# Supplementary material for: Diffusion spectrum imaging in patients with idiopathic normal pressure hydrocephalus: correlation with ventricular enlargement
Source: BMC Neurol. 2024 Jul 16;24:246. doi: 10.1186/s12883-024-03741-w (PMC11251323; doi:10.1186/s12883-024-03741-w)
Supplement: Supplementary file 1 — Supplementary Material 1 [file 12883_2024_3741_MOESM1_ESM.docx]

***Supplementary Materials 1***

Table S1. Successful recognition rate for each of the 18 tracts and their subcomponents in the idiopathic normal pressure hydrocephalus.

| Number | Tract | n | Ratio |
| --- | --- | --- | --- |
|  |  | N(iNPH)=32 | n/N(iNPH) |
| 1 | AF_L | 32 | 100% |
| 2 | AF_R | 31 | 96.9% |
| 3 | CST_L | 32 | 100% |
| 4 | CST_R | 31 | 96.9% |
| 5-6 | CC |  |  |
|  | Forceps Minor | 27 | 84.4% |
|  | Forceps Major | 31 | 96.9% |
| 7-8 | Bilateral Cingulum |  |  |
|  | C_FPH_L | 32 | 100% |
|  | C_FPH_R | 32 | 100% |
|  | C_PHP_L | 30 | 93.8% |
|  | C_PHP_R | 31 | 96.9% |
|  | C_PH_L | 32 | 100% |
|  | C_PH_R | 32 | 100% |
|  | C_FP_L | 32 | 100% |
|  | C_FP_R | 32 | 100% |
|  | Bilateral C_R | 22 | 68.8% |
| 9-10 | Bilateral UF |  |  |
|  | UF_L | 32 | 100% |
|  | UF_R | 32 | 100% |
| 11-12 | Bilateral SLF |  |  |
|  | SLF 1_L | 28 | 87.5% |
|  | SLF 1_R | 28 | 87.5% |
|  | SLF 2_L | 32 | 100% |
|  | SLF 2_R | 31 | 96.9% |
|  | SLF 3_L | 32 | 100.0% |
|  | SLF 3_R | 32 | 100.0% |
| 13-14 | Bilateral TR |  |  |
|  | TRA_L | 30 | 93.8% |
|  | TRA_R | 30 | 93.8% |
|  | TRP_L | 30 | 93.8% |
|  | TRP_R | 28 | 87.5% |
|  | TRS_L | 27 | 84.4% |
|  | TRS_R | 19 | 59.4% |
| 15 | ILF_L | 31 | 96.9% |
| 16 | ILF_R | 32 | 100.0% |
| 17 | IFOF_L | 30 | 93.8% |
| 18 | IFOF_R | 31 | 96.9% |

iNPH, idiopathic normal pressure hydrocephalus; AF, arcuate fasciculus; CST, corticospinal tract; CC, corpus callosum; C_FPH, cingulum frontal parahippocampal; C_FP, cingulum frontal parietal; C_PHP, cingulum parahippocampal parietal; C_PH, cingulum parahippocampal; C_R, cingulum rarolfactory; UF, uncinate fasciculus; SLF, superior longitudinal fasciculus; SLF 1, superior longitudinal fasciculus 1; SLF 2, superior longitudinal fasciculus 2; SLF 3, superior longitudinal fasciculus 3; TR, thalamic radiation; TRA, thalamic radiation anterior; TRP, thalamic radiation posterior; TRS, thalamic radiation superior; ILF, inferior longitudinal fasciculus; IFOF, inferior fronto-occipital fasciculus; L, left; R, right. n,represent the successfully recognized number of iNPH subjects in each tract; N(iNPH), number of total iNPH subjects
