# Supplementary material for: Diffusion spectrum imaging in patients with idiopathic normal pressure hydrocephalus: correlation with ventricular enlargement
Source: BMC Neurol. 2024 Jul 16;24:246. doi: 10.1186/s12883-024-03741-w (PMC11251323; doi:10.1186/s12883-024-03741-w)
Supplement: Supplementary file 2 — Supplementary Material 2 [file 12883_2024_3741_MOESM2_ESM.docx]

***Supplementary Materials 2***


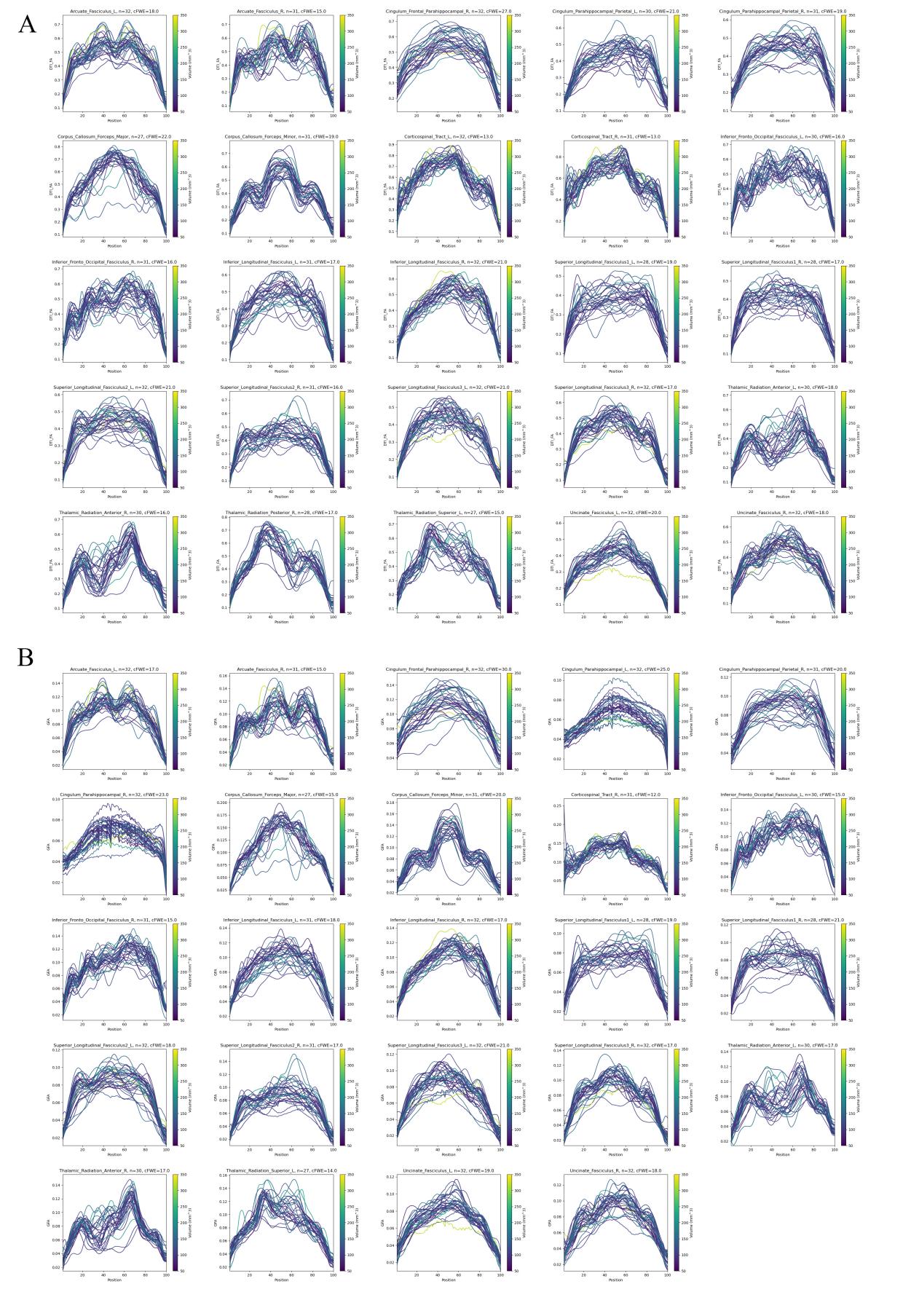


Figure S2. Other tracts analyzed along the 18 fibre tracts and relative subcomponents presented no significant correlation between FA or GFA and ventricular volume. Each line represents the subject analyzed, and different line colors indicate different lateral ventricular volumes,Colorbar from 50 ml (purple) to 350 ml (yellow). iNPH, idiopathic normal pressure hydrocephalus; L, left; R,right; FA, fractional anisotropy; GFA, general fractional anisotropy; n (iNPH), successfully recognized number of patients with iNPH in these fibers.(A) Correlation analysis between ventricular volume and FA of fibre tracts including the arcuate fasciculus, corticospinal tract, corpus callosum forceps minor, corpus callosum forceps major,cingulum frontal parahippocampal, cingulum parahippocampal parietal, superior longitudinal fasciculi 1, 2, and 3, thalamic radiation anterior, thalamic radiation posterior, thalamic radiation superior, inferior longitudinal fasciculus, and inferior fronto-occipital fasciculus, uncinate fasciculus. L, left; R, right. (B) Correlation analysis between ventricular volume and GFA of fibre tracts including the arcuate fasciculus, corticospinal tract, corpus callosum forceps minor,corpus callosum forceps major, cingulum frontal parahippocampal, cingulum parahippocampal, cingulum parahippocampal parietal, superior longitudinal fasciculi 1, 2, and 3, thalamic radiation anterior, thalamic radiation posterior, thalamic radiation superior, inferior longitudinal fasciculus, and inferior fronto-occipital fasciculus,uncinate fasciculus.
